# On the Differentiation of Auto- and Allo-Anti-D: The Significance of DAT and Autocontrol

**DOI:** 10.22034/ijp.2026.2086776.3636

**Published:** 2026-08-01

**Authors:** Alireza Abdollahi, Samaneh Salarvand

**Affiliations:** 1 Department of Pathology, School of Medicine, Imam Khomeini Hospital Complex, Tehran, Iran; 2 Associate Editor, Iranian Journal of Pathology, Tehran, Iran

## Dear Editor,

Anti-D is typically interpreted as an alloantibody in RhD-negative individuals after transfusion or pregnancy (1, 2). In contrast, detection of anti-D in an RhD-positive patient is uncommon and creates an important diagnostic challenge: true auto-anti-D versus allo-anti-D in the setting of a partial or variant D phenotype. We report two recent cases with anti-D reactivity but different immunohematologic mechanisms (3, 4).

Case 1 was a 49-year-old man with diabetes mellitus and thalassemia minor who presented with hypotension and anemia (hemoglobin, 9.5 g/dL). Initial ABO/RhD typing was O, RhD-positive (anti-D, 3+). Antibody screening with IMMUCOR screening reagent cells (USA) was reactive with screening cells 1 and 2 at all phases (room temperature [RT], albumin, and antihuman globulin [AHG]; approximately 2+, 3+, and 4+, respectively), while cell 3 was nonreactive. Importantly, both the direct antiglobulin test (DAT) and autocontrol were strongly positive (3+). Laboratory markers of hemolysis, such as lactate dehydrogenase and bilirubin, were not consistent with immune-mediated hemolysis. Because the reactive panel cells were D-positive and the D-negative panel cell was nonreactive, anti-D specificity was considered. However, the concurrent positive DAT and autocontrol supported an autoantibody pattern rather than classical alloimmunization. The sample was referred to the Blood Transfusion Organization for confirmatory testing. Additional serologic evaluation, including elution studies, confirmed the presence of auto-anti-D.

Case 2 was a 46-year-old woman with acute B-lymphoblastic leukemia who was typed as group A, RhD-negative. Her antibody screen showed reactivity with cells 1 and 2 (D-positive) and nonreactivity with cell 3 (D-negative), again suggesting anti-D specificity. After prewarming/warm saline methods, RT reactivity was removed, but reactions persisted in the albumin/AHG phases. In this case, the DAT and autocontrol were negative, supporting allo-anti-D rather than auto-anti-D.

These cases emphasize that anti-D specificity alone does not define the immunologic category of the antibody. A practical distinction requires integration of (1) RhD phenotype, (2) the pattern of reactivity against D-positive versus D-negative reagent cells, (3) DAT/autocontrol results, and (4) phase/thermal characteristics (5, 6). In RhD-positive patients, anti-D should not be dismissed as an error; two biologically plausible explanations remain: auto-anti-D and allo-anti-D due to partial D or other RHD variants. A positive DAT/autocontrol, as in our first case, strongly favors autoimmunity; nevertheless, complementary evaluations are essential to confirm the diagnosis. By contrast, a negative DAT/autocontrol with D-specific panel reactivity, as in our second case, is more consistent with alloimmunization (7, 8).

From a transfusion safety perspective, this distinction is clinically relevant. Patients with allo-anti-D should receive RhD-negative red cells (2, 5). Patients with suspected auto-anti-D may require phenotype-matched or least-incompatible support, depending on urgency and local policy, with close clinical and laboratory monitoring for hemolysis. For RhD-positive patients in whom anti-D is detected, RHD genotyping should be considered, when available, to identify partial or variant D and refine long-term transfusion and obstetric strategies (1, 3). In conclusion, anti-D reactivity in RhD-positive individuals should trigger a structured workup rather than immediate labeling as an alloantibody. Combining serologic pattern analysis with DAT/autocontrol interpretation can rapidly distinguish autoimmune from alloimmune mechanisms and improve transfusion decisions.

## Data Availability

All data generated or analyzed during this study are included in this published article.
